# Differential splicing of the apoptosis-associated speck like protein containing a caspase recruitment domain (ASC) regulates inflammasomes

**DOI:** 10.1186/1476-9255-7-23

**Published:** 2010-05-18

**Authors:** Nicole B Bryan, Andrea Dorfleutner, Sara J Kramer, Chawon Yun, Yon Rojanasakul, Christian Stehlik

**Affiliations:** 1 Division of Rheumatology, Department of Medicine and Robert H. Lurie Comprehensive Cancer Center, Feinberg School of Medicine, Northwestern University, 240 E. Huron St., Chicago, IL 60611, USA; 2 Program in Cancer Cell Biology, Health Sciences Center, West Virginia University; 1 Medical Center Drive, PO Box 9300, Morgantown, WV 26506, USA; 3 Department of Basic Pharmaceutical Sciences, School of Pharmacy, Health Sciences Center, West Virginia University, 1 Medical Center Drive, PO Box 9530, Morgantown, WV 26506, USA

## Abstract

**Background:**

The apoptotic speck-like protein containing a caspase recruitment domain (ASC) is the essential adaptor protein for caspase 1 mediated interleukin (IL)-1β and IL-18 processing in inflammasomes. It bridges activated Nod like receptors (NLRs), which are a family of cytosolic pattern recognition receptors of the innate immune system, with caspase 1, resulting in caspase 1 activation and subsequent processing of caspase 1 substrates. Hence, macrophages from ASC deficient mice are impaired in their ability to produce bioactive IL-1β. Furthermore, we recently showed that ASC translocates from the nucleus to the cytosol in response to inflammatory stimulation in order to promote an inflammasome response, which triggers IL-1β processing and secretion. However, the precise regulation of inflammasomes at the level of ASC is still not completely understood. In this study we identified and characterized three novel ASC isoforms for their ability to function as an inflammasome adaptor.

**Methods:**

To establish the ability of ASC and ASC isoforms as functional inflammasome adaptors, IL-1β processing and secretion was investigated by ELISA in inflammasome reconstitution assays, stable expression in THP-1 and J774A1 cells, and by restoring the lack of endogenous ASC in mouse RAW264.7 macrophages. In addition, the localization of ASC and ASC isoforms was determined by immunofluorescence staining.

**Results:**

The three novel ASC isoforms, ASC-b, ASC-c and ASC-d display unique and distinct capabilities to each other and to full length ASC in respect to their function as an inflammasome adaptor, with one of the isoforms even showing an inhibitory effect. Consistently, only the activating isoforms of ASC, ASC and ASC-b, co-localized with NLRP3 and caspase 1, while the inhibitory isoform ASC-c, co-localized only with caspase 1, but not with NLRP3. ASC-d did not co-localize with NLRP3 or with caspase 1 and consistently lacked the ability to function as an inflammasome adaptor and its precise function and relation to ASC will need further investigation.

**Conclusions:**

Alternative splicing and potentially other editing mechanisms generate ASC isoforms with distinct abilities to function as inflammasome adaptor, which is potentially utilized to regulate inflammasomes during the inflammatory host response.

## Background

Inflammasomes are inducible multi-protein platforms in phagocytic cells that are required for activation of caspase 1 by induced proximity during the inflammatory host response following pathogen infection and tissue damage [1]. The best characterized substrates for caspase 1 are interleukin (IL)-1β and IL-18, two potent pro-inflammatory cytokines [2]. However, a number of alternative substrates have been recently identified [3,4]. While generation of bioactive IL-1β and IL-18 is regulated at multiple steps, including transcription, posttranslational processing and receptor binding [2], their maturation into the bioactive secreted 17 and 18 kDa forms is dependent on the proteolytically active caspase 1 [5,6]. Inflammasomes are activated in response to the recognition of damage-associated molecular patterns (DAMPs) derived from pathogens (PAMPs) or host (danger or stress signals) by members of the cytosolic Nod-like receptor (NLR) family of cytosolic pattern recognition receptors (PRRs) [6-10]. The largest subfamily of NLRs contains a PYRIN domain (PYD) as an effector domain [11]. Activated NLRs undergo NTP-dependent oligomerization in response to DAMP recognition, and recruit the essential adaptor protein ASC by PYD-PYD interaction [12,13]. ASC subsequently bridges to caspase 1 through caspase recruitment domain (CARD)-CARD interaction [14,15]. Macrophages with ASC gene deletion are impaired in their ability to form inflammasomes and activate caspase 1 in response to a number of DAMPs, underscoring the critical role of ASC as an adaptor protein linking activated NLRs to caspase 1 [16-18]. Recently, pyrin has also been implicated in assembling an inflammasome, and the cytosolic DNA sensor AIM2 forms a caspase 1 activating inflammasome, too [19-23].

IL-1β and IL-18 have a central role in the inflammatory host response. However, dysregulation of the inflammasome complex causes their uncontrolled and excessive secretion, and is directly linked to an increasing number of human inflammatory diseases. NLRP1 polymorphisms are linked with autoimmune diseases that cluster with vitiligo, including autoimmune thyroid disease, latent autoimmune diabetes, rheumatoid arthritis, psoriasis, pernicious anemia, systemic lupus erythematosus, and Addison's disease [24]. NLRP3-containing inflammasomes are linked to contact hypersensitivity, sunburn, essential hypertension, gout and pseudogout, Alzheimer's disease, and elevated expression of NLRP3 is detected in synovial fluids of RA patients [25-30]. Furthermore, hereditary mutations in NLRP3 rendering the protein constitutively active, are directly linked to cryopyrin-associated periodic syndromes (CAPS) [31,32]. Hereditary mutations in pyrin, the causative for Familial Mediterranean fever (FMF) and in PSTPIP1, a pyrin interacting protein responsible for Pyogenic arthritis, pyoderma gangrenosum, and acne syndrome (PAPA), are responsible for impaired regulation of IL-1β maturation [33-35]. Mutant NLRP3 proteins efficiently form complexes with ASC to mediate caspase 1 activation independent of an activating ligand. This finding demonstrates the potential benefits of controlling the recruitment of ASC to NLRs.

Several molecular mechanisms have been linked to control inflammasome activation, including single PYD or CARD-containing proteins, pyrin and some NLRs [36]. We recently demonstrated that upon infections and cell stress conditions, such as treatment of cells with bacterial RNA or heat killed gram positive and gram negative bacteria, ASC redistributes from the nucleus to the cytosol, where it aggregates with NLRs and caspase 1 into perinuclear structures [37]. Sequestering ASC inside the nucleus completely prevented caspase 1 activation and processing and release of IL-1β, suggesting that redistribution of ASC might function as a check-point to prevent spontaneous and unwanted inflammasome activation.

Here we report on the identification of three ASC isoforms with distinct abilities to function as inflammasome adaptor, suggesting that differential splicing of the ASC pre-mRNA might potentially modulate the inflammatory host responses at the level of inflammasomes.

## Methods

### Materials and Reagents

Monoclonal ASC-PYD-specific antibodies were from MBL (D086-3, clone 23-4, 1:1000), rabbit polyclonal ASC-PYD-specific antibodies recognizing mouse ASC were from Alexis (AL177, 1:500) and ASC-CARD-specific antibodies were from Chemicon (AB3607, 1:500), and rabbit polyclonal ASC-Linker-specific antibodies were custom raised (CS3 1:10,000) using the peptide CGSGAAPAGIRAPPQSAAKPG corresponding to amino acids 93-111 of human ASC [37].

### Expression Plasmids

A search of the publicly available expressed sequence tag (EST) database revealed three potential ASC isoforms: ASC-b (Acc. No. BM456838), ASC-c (Acc. No. BE560228), and ASC-d (Acc. No. BM920038). The complete open reading frame of each isoform was subsequently amplified by PCR from pooled THP-1 cell cDNAs that were induced with a cocktail of cytokines for 2 to 24 hours. ASC-b, ASC-c, and ASC-d were amplified using the common forward primer 5'-CGGAATTCGATCCTGGAGCCATGGG-3' and the common reverse primer 5'-CGCTCGAGTGACCGGAGTGTTGCTG-3' and cloned into a modified pcDNA3 vector (Invitrogen) in frame with an NH_2_-terminal myc epitope tag. The CARD of caspase 1 was amplified by high fidelity PCR and cloned into pGex4T-1 (Novagen). All other expression constructs (ASC, pro-IL-1β, pro-caspase 1, NLRP3^R260W^) have been previously described [37-39].

### RT-PCR

THP-1 cells were differentiated into adherent macrophages by o/n culture in complete medium supplemented with 25 ng/ml phorbol 12-myristate 13-acetate (PMA; Calbiochem) and further cultured for 2 days, followed by treatment with LPS as indicated. Total RNA was isolated using Trizol (Invitrogen), reverse transcribed into cDNA (Superscript III, Invitrogen) and analyzed for ASC mRNA expression by RT-PCR using the following primer pairs: pr-1: 5'-GCTGTCCATGGACGCCTTGG-3', 5'-CATCCGTCAGGACCTTCCCGT-3' (ASC: 299 bp, ASC-b: 242 bp); pr-2: 5'-GCCATCCTGGATGCGCTGGAG-3', 5'-GGCCGCCTGCAGCTTGAAC-3' (ASC-c: 66 bp); pr-3: 5'-CTGACCGCCGAGGAGCTCAAGAAGT-3', 5'-GGCGCCGTAGGTCTCCAGGTAGAAG-3' (ASC and ASC-b: 128 bp, ASC-d: 100 bp); β actin 5'-GGATGGCATGGGGGAGGGCATA-3', 5'-TGATATCGCCGCGCTCGTCGTC-3' (533 bp).

### Cell Culture

HEK293, RAW264.7, THP-1 and J774A1 cells were obtained from the American Type Culture Collection (ATCC) and maintained in DMEM supplemented with 10% FBS, 4 mM L-glutamine, 0.1 mM non-essential amino acids, 1 mM sodium pyruvate, 1.5 g/L sodium bicarbonate, and 1% penicillin/streptomycin antibiotics (HEK293, RAW264.7, J774A1) or RPMI medium (ATCC) containing 2 mM L-glutamine, 10 mM HEPES, 1 mM sodium pyruvate, 4500 mg/l glucose, supplemented with 1500 mg/l sodium bicarbonate, 0.05 mM 2-mercaptoethanol and 10% FBS (THP-1). Human peripheral blood mononuclear cells (PBMC) were isolated by Ficoll-Hypaque centrifugation (Sigma) from buffy coats obtained from healthy donors and countercurrent centrifugal elutriation in the presence of 10 μg/ml polymyxin B sulfate using a JE-6B rotor (Beckman Coulter). PBMC were washed in Hank's Buffered Salt Solution, resuspended in serum-free DMEM for 1 hour and then cultured in complete medium supplemented with 20% FBS for 7 days to differentiate peripheral blood macrophages (PBM). HEK293 cells were transiently transfected using Polyfect (Qiagen) or Xfect (Clontech) according the procedures recommended by the manufacturer.

### Stable Cells

RAW264.7 were stably transfected with linearized expression vectors using the Amaxa Nucleofector using program H-033, 2 × 10^6 ^cells and 1.75 μg DNA, and selected with 1 mg/ml G418 for 14 days and tested for expression by immunoblot and immunofluorescence. Stable ASC-c expressing THP-1 and J774A1 cells were generated by lentivirus transduction. ASC-c was shuttled into the pLEX expression plasmid (Open Biosystems) modified to contain Myc or GFP epitope tags. Lentivirus was produced by co-expression of pLEX with pMD2.G and psPAX2 (Addgene plasmids 12259 and 12260) in 12-well dishes and 250 μl clarified culture supernatant was used to transduce 10^5 ^THP-1 and J774A1 cells using 4 μg/ml Polybrene and the ExpressMag transduction enhancing system (Sigma) in 96-well dishes for 4 hours at 32°C, followed by Puromycin selection.

### Immunofluorescence

HEK293 cells were seeded onto Type I collagen-coated (5 μg/cm^2^) glass cover slips in 6-well plates. The following day they were transfected with plasmids encoding each of the ASC isoforms alone or co-transfected with GFP-NLRP3^R260W^, GFP-pro-caspase 1^C285A^, or HA-tagged ASC. 36 hours post-transfection, cells were fixed in 3.7% paraformaldehyde, incubated in 50 mM glycine for 5 minutes and permeabilized and blocked with 0.5% saponin, 1.5% BSA, 1.5% normal goat serum for 30 minutes. Immunostaining was performed with polyclonal anti-myc or HA antibodies (Santa Cruz Biotechnology, 1:400) or monoclonal anti-myc antibodies (Santa Cruz Biotechnology, 1:400; Northwestern University Monoclonal Antibody Facility, 1:10,000). Secondary Alexa Fluor 488 and 546-conjugated antibodies, Topro-3, DAPI, and phalloidin were from Molecular Probes. Cells were washed with PBS containing 0.5% saponin, and cover slips were mounted using Fluoromount-G (Southern Biotech). Images were acquired by confocal laser scanning microscopy on a Zeiss LSM 510 Meta and epifluorescence microscopy on a Nikon TE2000E2 with a 100× oil immersion objective and image deconvolution (Nikon Elements). Presented are representative results observed in the majority of cells from several repeats.

### Subcellular fractionation

10^6 ^cells were resuspended in hypotonic lysis buffer (10 mM Tris-HCL pH 7.4, 10 mM NaCl, 3 mM MgCl_2_, 1 mM EDTA, and 1 mM EGTA, supplemented with protease and phosphatase inhibitors), incubated on ice, adjusted to 250 mM sucrose, and lysed using a Dounce homogenizer. Samples were initially centrifuged at 4°C at 1,000 × g for 3 minutes to remove any intact cells and then centrifuged at 4°C at 2,000 × g for 10 minutes to pellet the nuclei. The cytosolic supernatant was removed, and the nuclear pellet was then washed three times in hypotonic lysis buffer with the addition of 250 mM sucrose and 0.1% NP-40 and incubated for 20 minutes on ice. Both fractions were adjusted to 50 mM Tris-HCl pH 7.4, 20 mM NaCl, 3 mM MgCl_2_, 250 mM sucrose, 0.5% deoxycholate, 0.1% SDS, 0.2% NP-40, and protease and phosphatase inhibitors, and fully solubilized by brief sonication. 50 μg of protein lysates were separated by SDS-PAGE, transferred to a PVDF membrane, and probed with anti-ASC antibodies and HRP-conjugated secondary antibodies (Amersham Pharmacia) in conjunction with an ECL detection system (Pierce). Membranes were stripped and re-probed with anti-GAPDH (Sigma) and anti-Lamin A (Santa Cruz Biotechnology) antibodies as control for cytosolic and nuclear fractions, respectively.

### Measurement of IL-1β secretion

HEK293 cells were seeded into type-I collagen-coated 12-well dishes, and allowed to attach overnight. Cells were co-transfected in triplicates the following day with expression constructs encoding the constitutively active NLRP3^R260W ^(0.675 μg), pro-caspase 1 (0.15 μg), and mouse pro-IL-1β (0.375 μg), and each of the ASC isoforms ASC-b, -c, or -d (0.04 μg) or ASC (0.015 μg), either alone or in the presence of full-length ASC to reconstitute inflammasomes. The total amount of DNA was kept constant with the addition of an empty pcDNA3 vector as necessary. The media was replaced 24 hours post-transfection, and at 48 hours post transfection, the supernatants were collected, clarified by centrifuged at 13,000 rpm for 15 minutes at 4°C, and analyzed by ELISA for mouse IL-1β release according to the manufacturer's protocol (BD Biosciences). RAW 264.7^Ctrl^, RAW 264.7^ASC^, RAW 264.7^ASC-b^, J774A1^Ctrl^, J774A1^ASC-c^, THP-1^Ctrl^, THP-1^ASC-C#1^, and THP-1^ASC-c#2 ^cells were seeded into 24-well dishes and either left untreated or treated with 300 ng/ml LPS (E. coli, 0111:B4) for 16 hours followed by the collection of culture supernatants (THP-1 cells), or followed by pulsing with ATP (5 mM for RAW264.7 and 3 mM for J774A1 cells) for 15 minutes and collection of culture supernatants. Clarified culture supernatants were analyzed for secreted mouse (RAW264.7, J774A1) or human (THP-1) IL-1β by ELISA (BD Biosciences) according to the manufacturer's protocol.

### In vitro protein-interaction assay

ASC and ASC-b were *in vitro *translated and biotinylated using the TNT Quick Coupled Transcription/Translation system (Promega) according to the manufacturer's protocol. GST-caspase 1-CARD was affinity purified from *E. coli *BL21, following induction with 1 mM IPTG for 4 hours at room temperature. Cells were resuspended in STS buffer (10 mM Tris pH 8.0, 1 mM EDTA, and 150 mM NaCl), lysed by several rapid freeze/thaw cycles followed by the addition of lysozyme (1 mg/ml). After a 30 minute incubation on ice, 10 mM DTT and 1.4% sodium sarkosyl were added, sonicated and cleared by centrifugation at 13,000 rpm for 15 minutes. Cleared lysates were adjusted to 4% Triton X-100 and incubated with immobilized glutathione sepharose (Pierce) overnight at 4°C. Beads were washed three times with 0.1% Triton X-100 in PBS, blocked for 30 minutes at room temperature in HKMEN buffer (142.4 mM KCl, 5 mM MgCl_2_, 10 mM HEPES (pH 7.4), 0.5 mM EGTA, 1 mM EDTA, 0.2% NP-40, 1 mM DTT) supplemented with protease inhibitors and BSA (1 mg/ml). Following one wash with HKMEN buffer, beads were incubated overnight on a rotator with *in vitro *translated ASC and ASC-b. Bound proteins were washed 4 times in HKMEN buffer supplemented with protease inhibitors, boiled in Laemmli buffer, separated by SDS/PAGE, transferred onto a PVDF membrane, and detected with Streptavidin-HRP in conjugation with an enhanced chemiluminescent reagent (Millipore).

## Results

### Identification of three novel ASC transcripts

**Figure 1:**
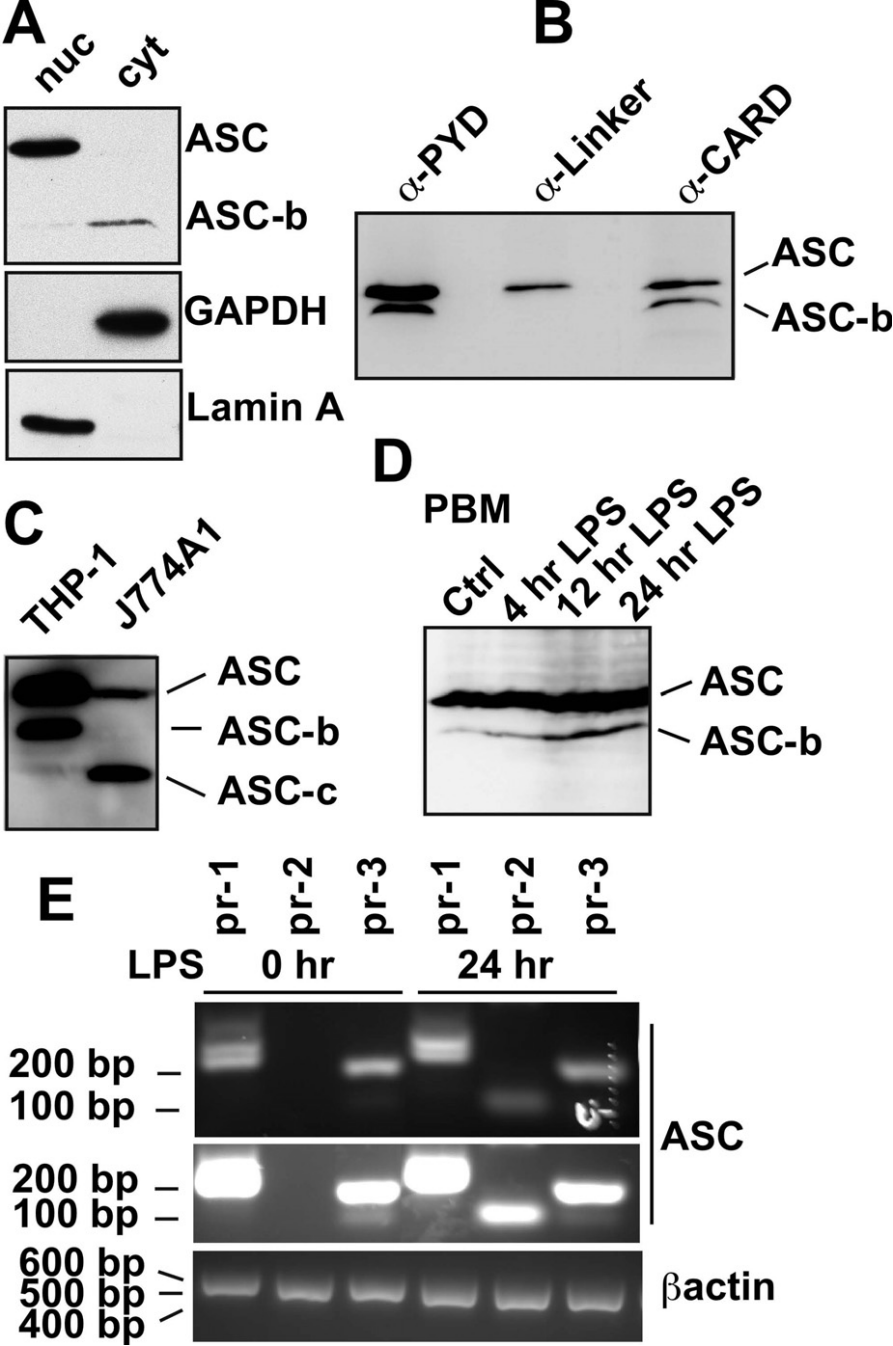
**Identification of ASC isoforms**. **(A) **Differentiated THP-1 macrophages were separated into nuclear and cytosolic fractions and analyzed for ASC expression using a monoclonal anti-ASC antibody recognizing the PYD of ASC by immunoblot. Blots were stripped and re-probed with antibodies for the cytosolic GAPDH and nuclear Lamin A to control for fractionation efficiency. **(B) **THP-1 lysates were analyzed by immunoblot for ASC expression using antibodies recognizing the PYD, the linker, and the CARD, respectively. **(C) **Lysates from PMA-differentiated and LPS-treated (300 ng/ml) THP-1 cells and J774A1 cells were separated by SDS/PAGE and immunoblotted with a PYD-specific anti-ASC antibody (AL177). **(D) **Lysates of human peripheral blood macrophages (PBM) that were left untreated, or treated with LPS for the indicated times, were immunoblotted for ASC. **(E) **PMA-differentiated THP-1 cells were treated with LPS (300 ng/ml) for the indicated times and analyzed by RT-PCR for ASC transcripts using the primer pairs pr-1 (ASC, 299 bp; ASC-b, 242 bp), pr-2 (ASC-c, 66 bp), and pr-3 (ASC and ASC-b, 128 bp; ASC-d, 100 bp). A short exposure (upper panel) and long exposure (middle panel) is shown, because of the relative low abundance of ASC-d transcripts. A β -actin primer pair (533 bp, lower panel) was used as a control.

We recently demonstrated that ASC localizes to the nucleus of resting macrophages and that inflammatory activation causes the inducible redistribution of ASC to the cytosol [37]. We consistently noted that a monoclonal ASC specific antibody directed to the PYD of ASC also specifically recognizes a protein with slightly lower molecular weight in the cytosol also in resting macrophages, which we named ASC-b (Figure 1A). The molecular weight appeared too large to correspond to one of the PYD-only proteins (POPs), which others and we identified as negative regulators of inflammasomes, and especially POP1 shares a high sequence similarity with ASC [36,38-42]. We used a panel of commercially available ASC specific antibodies that are directed to either the PYD or the CARD, and raised a custom polyclonal antibody to the linker domain to further characterize this protein. Using this strategy, we identified that the smaller protein is recognized by PYD and CARD specific antibodies, but that our linker specific antibody fails to detect the smaller protein in total protein lysates of THP-1 cells, suggesting that the linker that connects the PYD and the CARD in ASC is lacking in the smaller protein (Figure 1B). Furthermore, a polyclonal antibody raised against amino acid residues 2 to 27 of the PYD of ASC also detects ASC and ASC-b in lysates of PMA-differentiated THP-1 cells and an additional low abundant protein, which we named ASC-c (Figure 1C). This antibody also detects ASC in mouse J774A1 macrophages, which appear to lack ASC-b, but express significant levels of a putative ASC-c (Figure 1C). Also human peripheral blood macrophages (PBM) express ASC-b, which is upregulated following LPS treatment (Figure 1D). We did not detect ASC-c under the tested conditions, but PBM express significant lower ASC levels compared to THP-1 cells, and thus ASC-c might have gone undetected. *ASC *is encoded from three exons, and we therefore mined the publicly available EST database to potentially identify ASC alternative transcripts. We identified three distinct transcripts of ASC in addition to the full-length transcript expressed in human tissues. Based on these sequences, we designed specific PCR primers, and amplified all three cDNAs from a pooled human THP-1 cell cDNA library. We referred to these cDNAs as ASC-b, ASC-c, and ASC-d. ASC-b was already annotated within the NCBI GenBank and has recently been characterized as vASC by Matsushita and colleagues during the preparation of our manuscript [43]. We confirmed existence of these transcripts by RT-PCR using total RNA isolated from THP-1 cells, which we differentiated into adherent macrophage-like cells by incubation with PMA and treatment with LPS. In resting cells transcripts for ASC, ASC-b, and very low transcript numbers of ASC-d were present. LPS treatment caused the appearance of ASC-c (Figure 1E), suggesting that the presence of distinct combinations of ASC splice variants might potentially affect inflammasome activity at different stages of the inflammatory response.

**Figure 2:**
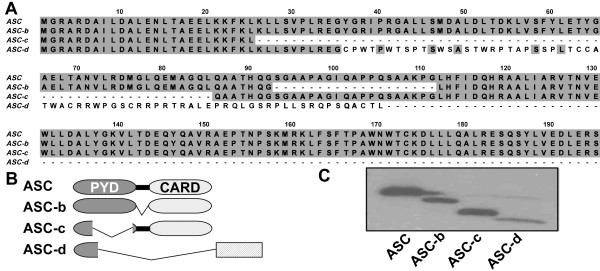
**Three novel ASC isoforms**. **(A) **Clustal W alignment of ASC, ASC-b, ASC-c and ASC-d. ASC consists of a PYD, linker, and CARD, while ASC-b displays an in frame deletion of amino acids 93 to 111, corresponding to the complete linker region. ASC-c lacks amino acids 26 to 85 corresponding to helices 3 to 6 of the ASC-PYD, and in ASC-d amino acids 36-195 are replaced with 69 unrelated amino acids due to a frame shift resulting in the deletion of nucleotides 107 to 134 in ASC-d. **(B) **Schemata showing the domain structure of the ASC isoforms. **(C) **Myc-tagged ASC, ASC-b, ASC-c and ASC-d were transiently transfected into HEK293 cells and expression of ASC proteins with the predicted molecular weight was verified by immunoblot using anti-myc antibodies.

ASC-b lacks amino acids 93 to 111, corresponding to the entire linker region, resulting in a protein with a directly fused PYD and CARD (Figure 2A, B). ASC-c lacks amino acids 26 to 85 corresponding to helices 3 to 6 of the ASC-PYD, but retains an intact ASC-Linker-CARD region (Figure 2A, B). ASC-d lacks nucleotides 107 to 134, which causes a frame shift and results in a protein consisting of helices 1 and 2 (amino acids 1-35) of the ASC-PYD fused to a novel 69 amino acid peptide without recognizable homology to any other known protein (Figure 2A, B). ASC and the three alternative cDNAs encode proteins of the predicted molecular weight, when expressed in HEK293 cells (Figure 2C). The ASC proteins that are abundantly expressed in THP-1 cells and are recognized by the ASC specific antibodies directed towards the PYD and CARD of ASC are ASC and ASC-b, while mouse J774A1 macrophages predominantly express ASC and a putative ASC-c.

At least two of the three alternative transcripts, ASC-b and ASC-c are likely generated through alternative mRNA splicing. The linker is encoded on exon 2 and is flanked by splice donor and acceptor sites. ASC-c likely utilizes an alternative 3' and 5' splice site and contains a potential splice acceptor site and a less conserved splice donor site. Generation of ASC-d could involve RNA editing, but its relationship to ASC and its generation and function in inflammasome regulation will need further investigations, due to its limited homology to ASC.

### ASC, ASC-b, ASC-c and ASC-d display distinct localization patterns

**Figure 3:**
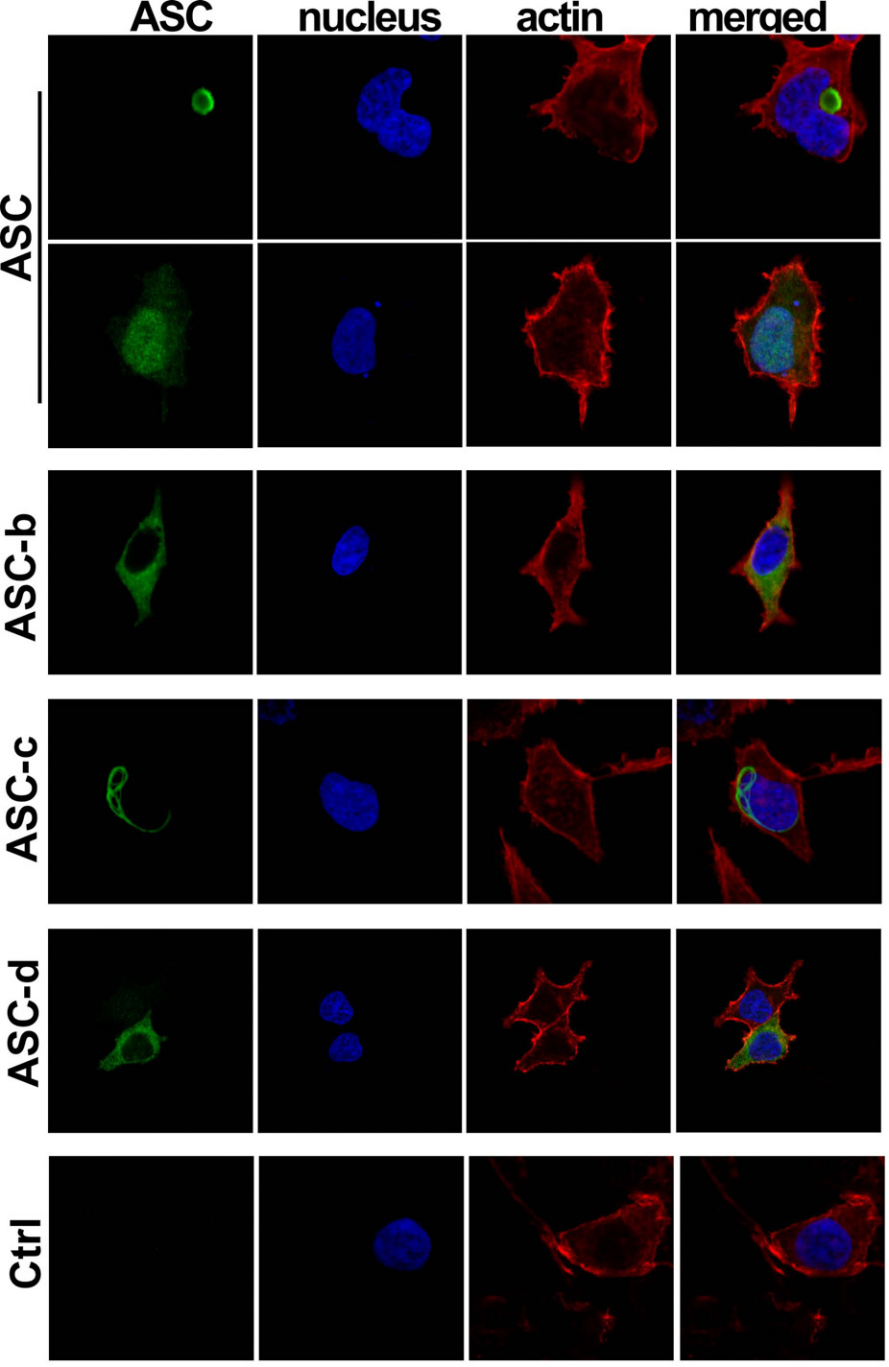
**Localization of ASC isoforms**. Subcellular localization of the myc-tagged ASC isoforms was examined in transiently transfected HEK293 cells. Cells were fixed and immunostained with monoclonal anti-myc antibodies and Alexa Fluor 488-conjugatetd secondary antibodies. Nuclei and actin were visualized using Topro-3 and Alexa Fluor 546-conjugated phalloidin, respectively. Images were acquired by laser scanning confocal microscopy, showing from left to right ASC (green), nucleus (blue), actin (red) and a merged composite image. The panels show ASC (1^st ^and 2^nd ^panels), ASC-b (3^rd ^panel), ASC-c (4^th ^panel), ASC-d (5^th ^panel), and vector control (6^th ^panel).

Ectopic expression of ASC displays a very characteristic localization pattern. It either localizes to the nucleus, diffusively throughout the cell, or to a perinuclear aggregate [44-46]. However, we recently demonstrated that this localization pattern is neither random nor caused by over expression of ASC, but that a similar distribution is also found for endogenous ASC, which is nuclear in resting macrophages, but is redistributed to cytoplasmic perinuclear aggregates in response to inflammatory activation of macrophages [37]. Therefore we investigated the localization patterns of the three alternate ASC proteins. Expression plasmids encoding each of the ASC isoforms were transiently transfected into HEK293 cells, and their subcellular distribution was analyzed by immunofluorescence microscopy. As previously reported, expression of full-length ASC resulted in the formation of the perinuclear aggregate (Figure 3, 1^st ^panel) or localization to the nucleus (Figure 3, 2^nd ^panel). However, none of the other isoforms retained the capacity to form these structures, but rather exhibited their own, unique localization pattern. ASC-b displayed a diffuse, exclusively cytoplasmic distribution (Figure 3, 3^rd ^panel), suggesting that either the linker is required for ASC self-aggregation and nuclear import, or some degree of flexibility provided by the linker is required for nuclear localization of ASC. ASC-c was also found exclusively in the cytoplasm. However, it oligomerized into long, filamentous structures, referred to as death filaments, which are also observed when the CARD or PYD of ASC is expressed by itself (Figure 3, 4^th ^panel) [47]. ASC-d localized primarily diffuse to the cytosol (Figure 3, 5^th ^panel). These results suggest that the linker region of ASC is required for efficient self-aggregation.

### ASC, ASC-b, ASC-c and ASC-d exhibit differences in their ability to co-localize with other inflammasome components

**Figure 4:**
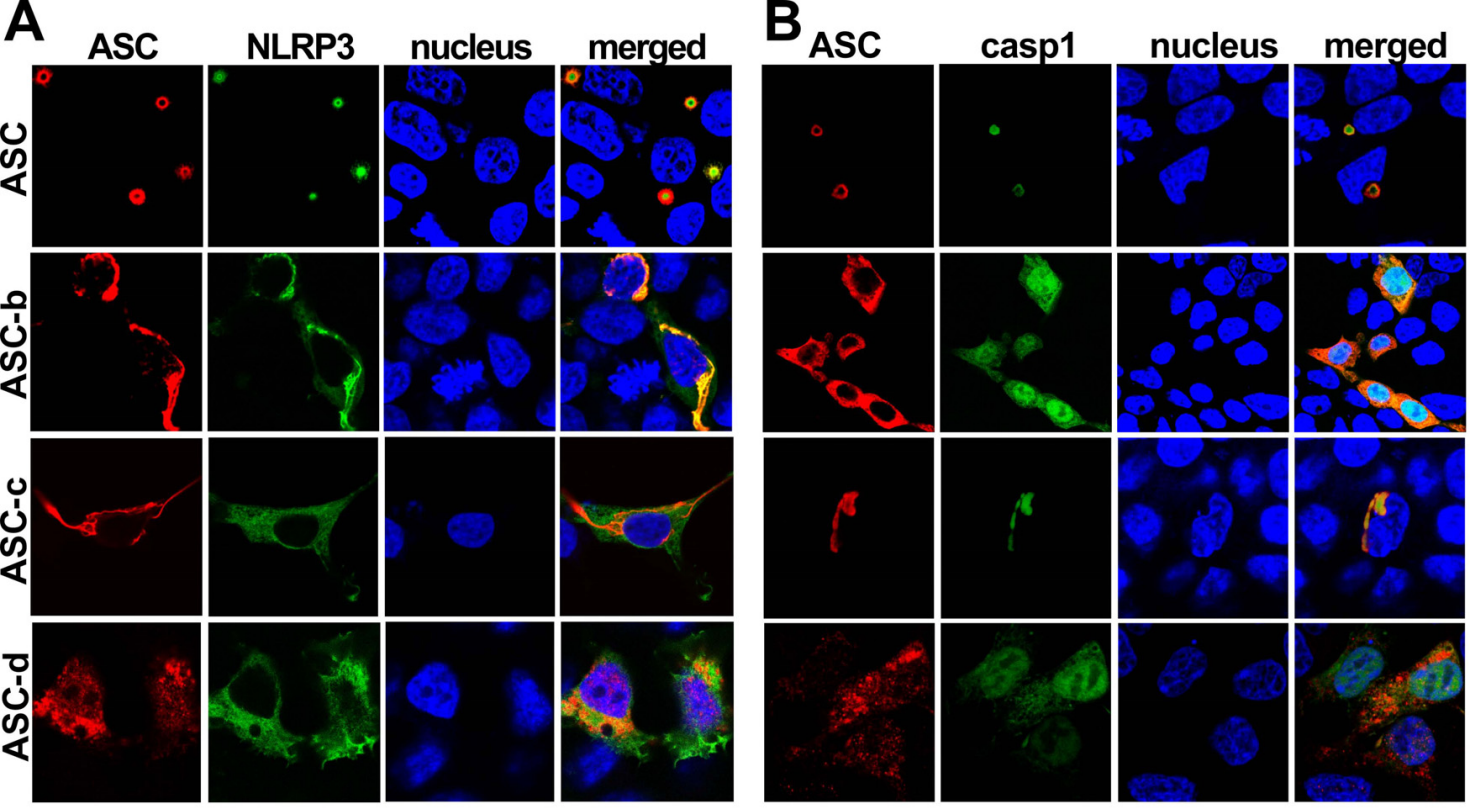
**Localization of ASC isoforms, NLRP3, and caspase 1**. ASC isoforms were transiently co-transfected into HEK293 cells with GFP-tagged NALP3^R260W ^(A) or GFP-tagged pro-caspase 1^C285A ^(B). Cells were fixed and immunostained with polyclonal anti-myc (Santa Cruz Biotechnology) and Alexa Fluor 546-conjugated secondary antibodies (Invitrogen). Topro-3 was used to visualize the nucleus. All images were acquired using laser scanning confocal microscopy with a 100x oil-immersion objective. Panels from left to right show ASC (red), NLRP3 or pro-caspase-1 (green), nucleus (blue), and a merged composite image.

ASC functions as an adaptor by interacting with NLRPs by PYD-PYD and with caspase 1 by CARD-CARD interaction, which are both essential to form inflammasomes, and all three proteins co-localize to aggregates [37]. We therefore tested the ability of the ASC isoforms to function as an inflammasome adaptor. HEK293 cells were co-transfected with a constitutively active GFP-tagged NLRP3^R260W ^mutant and each myc-epitope tagged ASC isoform, immunostained with myc-specific antibodies and analyzed for co-localization by immunofluorescence microscopy. As previously shown, full-length ASC and NLRP3^R260W ^co-localized in the perinuclear aggregates, when co-transfected (Figure 4A, 1^st ^panel). As expected, ASC-b, which still retains a fully intact PYD, also co-localized with NLRP3^R260W^. Co-expression of NLRP3 caused ASC-b to relocate from its diffuse cytosolic localization to form aggregates with NLRP3 (Figure 4, 2^nd ^panel). NLRP3 or NLRP3^R260W ^expression alone does not cause NLRP3 aggregation (data not shown). However, while these aggregates did exhibit a perinuclear localization, they were not as small and condensed as those observed with ASC. As expected due to lacking an intact PYD, neither ASC-c nor ASC-d was able to co-localize with NLRP3^R260W ^(Figure 4, 3^rd ^and 4^th ^panel).

Since ASC bridges NLRs with caspase 1, we next evaluated the capability of the ASC isoforms to interact with caspase 1. Because activation of caspase 1 would result in proteolytic cleavage of the CARD of pro-caspase 1, we expressed the C285A catalytically inactive mutant. We transiently co-transfected HEK293 cells with a GFP-pro-caspase 1^C285A ^fusion protein and each of the ASC isoforms, which were immunostained as above and analyzed by fluorescence microscopy. As previously shown, ASC did co-localize with caspase 1 into the characteristic aggregates (Figure 4B, 1^st ^panel) [15]. Also ASC-b, and ASC-c, which both contain an intact CARD, co-localized with pro-caspase 1, though this did not cause aggregation of ASC, suggesting that pro-caspase 1 is not sufficient to cause aggregation of ASC in the absence of an NLR (Figure 4B, 2^nd ^and 3^rd ^panel). ASC-b retained the diffuse cytosolic localization pattern that it exhibited when expressed alone. Furthermore, it only co-localized with cytoplasmic caspase 1, as it was excluded from the nucleus. ASC-c co-localized with caspase 1 in the long filamentous structures formed by ASC-c. In contrast, ASC-d did not co-localize with pro-caspase 1, as expected due to the lack of the CARD (Figure 4B, 4^th ^panel).

### ASC co-localizes with ASC-b and ASC-c

**Figure 5:**
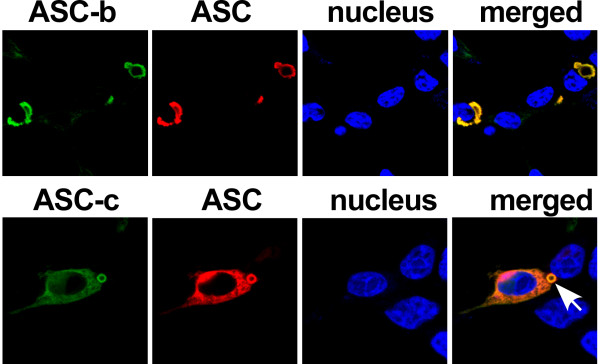
**Co-localization of ASC with ASC-b and ASC-c**. HEK 293 cells were transiently co-transfected with HA-tagged ASC and myc-tagged ASC-b (1^st ^panel) or ASC-c (2^nd ^panel). Cells were fixed and immunostained with monoclonal anti-myc (Millipore) and polyclonal anti-HA (Abcam) antibodies, and Alexa Fluor-488 and -546 conjugated secondary antibodies (Invitrogen), respectively. Topro-3 was used to visualize the nucleus. All images were acquired using laser scanning confocal microscopy with a 100× oil-immersion objective. Panels from left to right show ASC-b/ASC-c (green), ASC (red), nucleus (blue) and a merged composite image. An arrow points to the aggregate.

One of the mechanisms by which inflammasome assembly is regulated is through competitive PYD-PYD and CARD-CARD interactions between PYD-only proteins (POPs) or CARD-only proteins (COPs) with ASC, caspase 1 and NLRs [36]. Previous studies demonstrated that ASC can self-oligomerize via its CARD or PYD [40,48], we wanted to explore the possibility that the truncated ASC isoforms, ASC-b and ASC-c, could impair the inflammasome adaptor function of ASC. Co-expression of ASC with ASC-b resulted in the co-localization of both proteins in the perinuclear aggregates. However, the aggregates differed from those assembled by expression of ASC, and resulted in the formation of large, irregularly shaped perinuclear aggregates, rather than the small, circular structures formed by ASC a alone (Figure 5, 1^st ^panel). Co-expression of ASC with ASC-c also altered its subcellular localization pattern. Instead of the long filamentous structures formed by ASC-c, co-expression of ASC caused the recruitment of ASC-c to the perinuclear ASC aggregates. However, unlike those observed upon co-expression with ASC-b, these aggregates maintained all of the previously identified characteristics of ASC aggregates (Figure 5, 2^nd ^panel). However, there is also notably less efficient self aggregation of ASC in the presence of ASC-c, further suggesting that ASC-c potentially interferes with ASC oligomerization. These results indicate that the shorter isoforms can co-localize with ASC causing their recruitment to the ASC formed aggregate.

### Distinct ASC isoforms can either activate or inhibit inflammasome-mediated maturation of IL-1β

**Figure 6:**
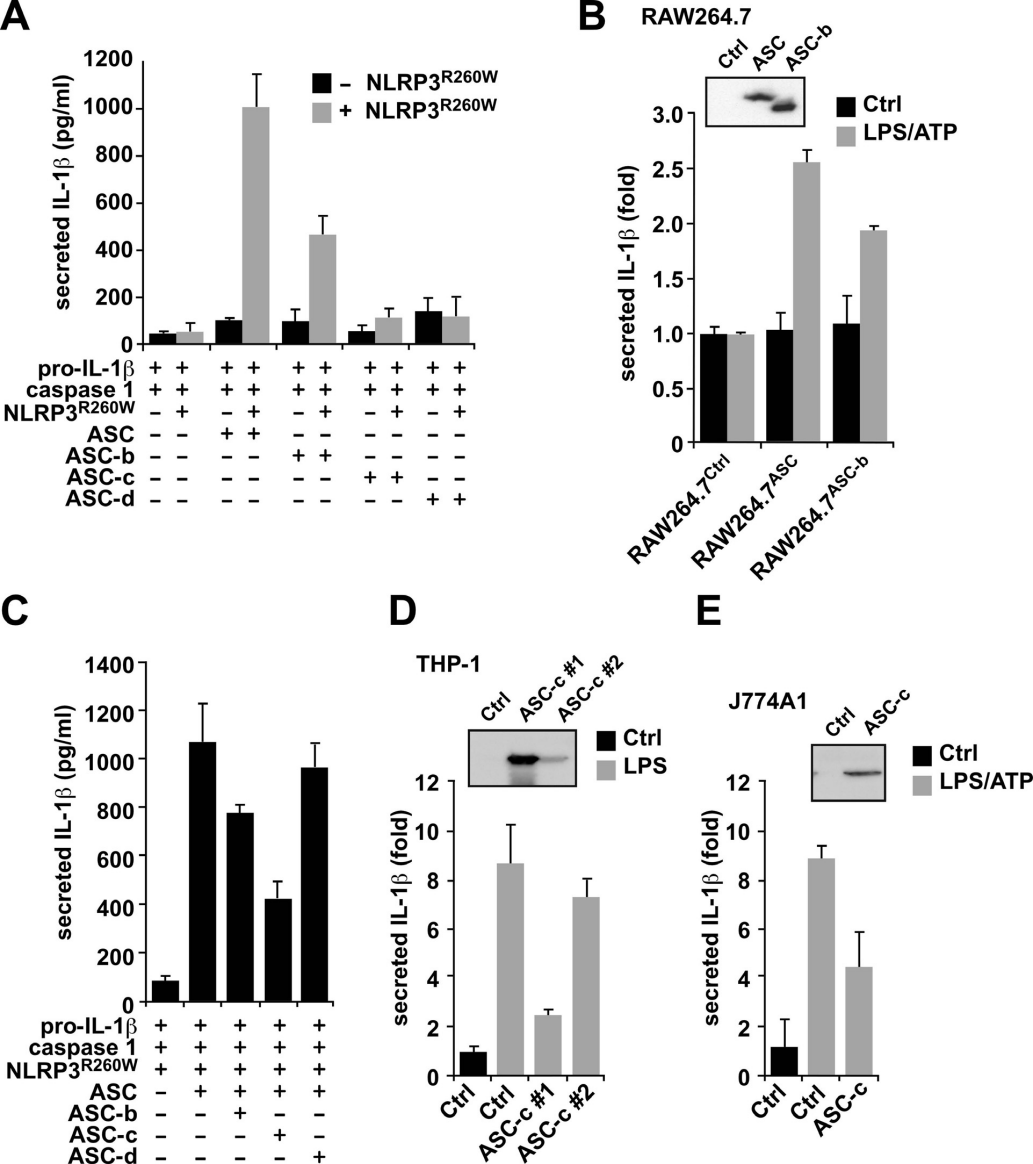
**Distinct ASC isoforms can function as either activating or inhibitory inflammasome adaptor**. **(A) **Inflammasomes were reconstituted in HEK293 cells by transient transfection of pro-IL-1β, pro-caspase 1, ASC, ASC-b, ASC-c, ASC-d, in the absence (black bars) or presence (gray bars) of the constitutive active NLRP3^R260W^, as indicated. Culture supernatants were analyzed for secreted IL-1β by ELISA 36 hours post transfection. **(B) **The ASC deficient RAW264.7 mouse macrophage cell line was stably transfected with empty vector, myc-tagged ASC, or myc-tagged ASC-b and analyzed for IL-1β release in resting cells (black bars) and following LPS (300 ng/ml)/ATP (5 mM) activation (gray bars). **(C) **Inflammasomes were reconstituted in HEK293 cells as shown in Figure 6A. Secreted IL-1β was analyzed by ELISA. All experiments were performed in triplicates (n = 3, +/- SD). **(D) **Control THP-1 cells (Ctrl) or THP-1 cells stably expressing high levels of ASC-c (#1) or low levels of ASC-c (#2) were treated with LPS (300 ng/ml) for 16 hours and analyzed for IL-1β release. Expression of ASC-c was determined by immunoblot. **(E) **Control J774A1 cells (Ctrl) or J774A1 cells stably expressing ASC-c were treated with LPS (300 ng/ml) for 16 hours, pulsed with 3 mM ATP for 15 minutes and analyzed for IL-1β release. Experiments in D and E were performed in triplicates (n = 2, +/- SD). Expression of ASC-c was determined by immunoblot. Note that the lysates from THP-1 and J774A1 cells were separated on the same gel and are the same exposure time.

Because ASC is essential for inflammasome formation and maturation and release of IL-1β in macrophages, we next determined how the different ASC isoforms impact inflammasome activity. We reconstituted NLRP3 inflammasomes in HEK293 cells, which lack endogenous expression of inflammasome components, but active inflammasomes can be formed by transient expression of the core inflammasome components [37-39]. Cells were transiently co-transfected with expression plasmids encoding pro-IL-1β, pro-caspase 1, and each of the ASC isoforms in the presence or absence of the constitutively active NLRP3^R260W^. Culture supernatants were collected thirty-six hours post-transfection and analyzed for released IL-1β by ELISA. Only ASC and ASC-b, which contain both the PYD and the CARD, were able to promote release of IL-1β into culture supernatants (Figure 6A). Lacking the linker domain reduced the ability of ASC-b to function as an inflammasome adaptor, although it contains the necessary PYD and CARD. As expected, neither ASC-c nor ASC-d was able to generate mature IL-1β.

The RAW 264.7 mouse macrophage cell line lacks ASC and is therefore deficient in the processing and release of IL-1β [49]. To test the two activating ASC isoforms under more physiological conditions, we stably transfected RAW264.7 cells with myc-tagged ASC, ASC-b, or an empty plasmid in an effort to restore the ASC deficiency in these cells. Control cells, ASC, and ASC-b stable cells were either left untreated or activated with LPS/ATP and culture supernatants were analyzed for secreted IL1β by ELISA. As previously shown, control cells did not process and release IL-1β in response to LPS and ATP. However, restoring ASC or ASC-b expression did result in a limited increase in IL-1β secretion in response to LPS/ATP, compared to resting cells (Figure 6B). As shown above in our inflammasome reconstitution system, also stable expression of ASC-b is less potent as inflammasome adaptor compared to ASC. Expression of ASC and ASC-b was confirmed by immunoblot using myc-specific antibodies (Figure 6B, insert).

Since we showed above that ASC-c and ASC-d are unable to function as inflammasome adaptor, but at least ASC-c is capable of co-localizing with caspase 1, we tested, whether ASC-c can interfere with the function of ASC as inflammasome adaptor by competing for caspase 1. We used the NLRP3 inflammasome reconstitution assay and transfected either ASC with empty vector, ASC-b, ASC-c, or ASC-d, in addition to pro-IL-1β, pro-caspase 1 and NLRP3^R260W^, and analyzed the culture supernatants for IL-1β as above. Co-transfection of ASC along with ASC-b caused a reduction of IL-1β release, likely because in some inflammasomes the less potent ASC-b is incorporated. As expected, co-transfection of ASC-c did significantly reduce IL-1β levels in the supernatant, suggesting that ASC-c might function similar as a CARD-only protein (COP). However, co-transfection of ASC-d did not significantly affect the previously characterized function of ASC, as determined by the similar levels of IL-1β detected in the supernatant (Figure 6C), indicating that generation of different isoforms of ASC have the potential to differentially regulate inflammasome activity. To further investigate the effect of ASC-c on inflammasome activity in a relevant cell system, we generated stable ASC-c expressing human THP-1 monocytic cell lines and mouse J774A1 macrophages by lentiviral transduction. THP-1^Ctrl ^and THP-1^ASC-c#1 ^(ASC-c high expressing cells) and THP-1^ASC-c#2 ^(ASC-c low expressing cells) were treated with LPS for 16 hours and culture supernatants were analyzed for IL-1β release. While THP-1^Ctrl ^cells robustly responded with IL-1β release to LPS treatment, THP-1^ASC-c#1 ^cells and to a much lesser extent THP-1^ASC-c#2 ^cells were impaired in IL-1β release, which inversely correlated with the expression levels of ASC-c, as shown by immunoblot (Figure 6D). J774A1 macrophages require LPS priming followed by ATP pulsing to secrete IL-1β. As observed for THP-1 cells, also J774A1^ASC-c ^cells, which express an intermediate level of ASC-c, showed diminished IL-1β release compared to J774A1^Ctrl ^cells following LPS priming and ATP pulsing (Figure 6E).

## Discussion

We report the existence of novel alternative isoforms of the essential inflammasome adaptor ASC, which have the potential to differentially regulate inflammasomes. They either promote (ASC, ASC-b), inhibit (ASC-c) or do not impact (ASC-d) inflammasome function. However, it still needs to be established, whether these alternative splice forms of ASC also contribute to inflammasome activity on endogenous level. Post-transcriptional modifications, such as alternative splicing, are common in genes regulating apoptotic and inflammatory pathways [50,51], and as much as 94% of all human genes undergo alternative splicing [52]. Alternative splicing of pre-mRNAs enables the production of multiple transcripts and proteins with distinct functions from a single gene. It has been observed for transcripts encoding several other inflammatory adaptor proteins, including the Nod adaptor RIP2 and the TLR/IL-1R adaptor MyD88, IRAK1 and IRAK2, and results in either activating or inhibitory effects on downstream signaling [53-56]. Based on annotated cDNA sequences and antibody mapping, we identified three novel isoforms of ASC, designated ASC-b, ASC-c, and ASC-d. Two of these isoforms are most likely generated through alternative splicing of the ASC pre-mRNA, while the mechanism giving rise to ASC-d remains unclear.

**Figure 7:**
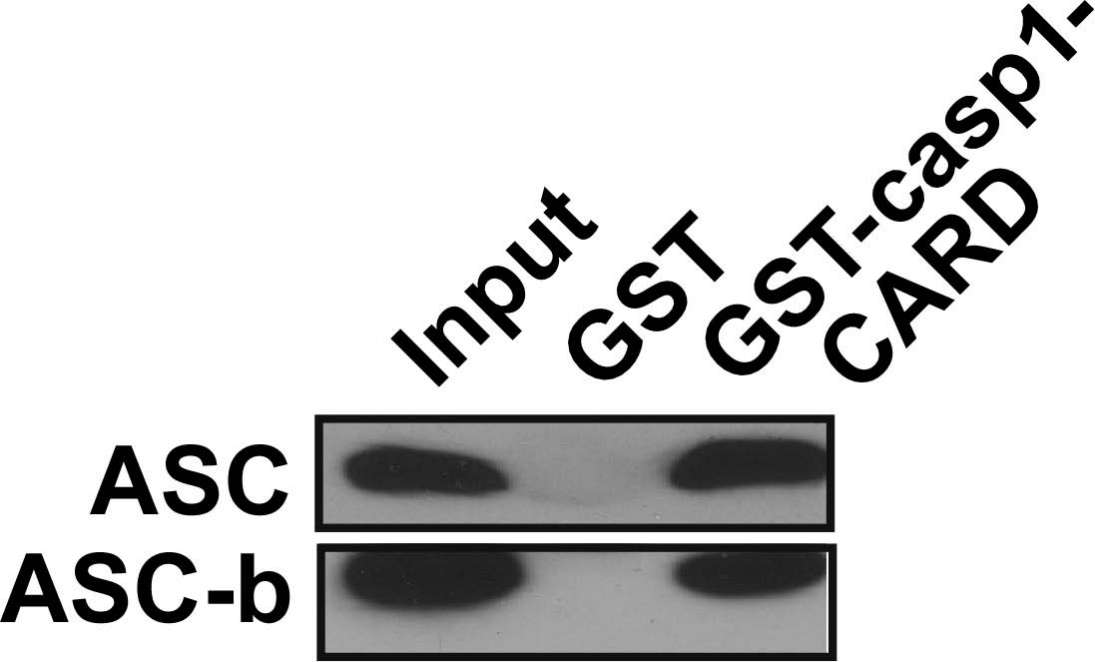
**ASC and ASC-b interact with pro-caspase 1 with similar affinity**. Immobilized GST-caspase 1-CARD was incubated with in vitro translated and biotinylated ASC or ASC-b and subjected to *in vitro *GST-pull down assays using GST-caspase 1-CARD and GST control immobilized to GSH Sepharose, as indicated. Bound proteins were visualized with streptavidin-HRP and ECL-Plus detection (Amersham Pharmacia Biotech). 10% of the in vitro translated proteins were loaded as 'input".

The significance of identifying different ASC isoforms generated by alternative splicing, is their different ability to function as inflammasome adaptor. While ASC shows the strongest activity as inflammasome adaptor, ASC-b shows reduced activity in gene transfer experiments and when restored in the ASC deficient RAW264.7 macrophage cell line, suggesting that the level of inflammasome activity can be regulated by availability of recruited ASC or ASC-b. ASC-b is commonly co-expressed with ASC and both function as inflammasome adaptor, though with different efficacy. Therefore the observed upregulation of ASC-b following prolonged LPS treatment in primary human macrophages can be expected to affect inflammasome activity. In our hands, ASC-b consistently displayed lower activity compared to ASC, while Matsushita and colleagues recently showed an increase in activity of ASC-b. This discrepancy might result from the system used to address the role of ASC versus ASC-b. Matsushita and colleagues tested activity of ASC and ASC-b by co-expressing pro-caspase 1, pro-IL-1β and either ASC or ASC-b. Our localization data demonstrated that both equally co-localize with caspase 1 and also interact to a similar extend, as determined biochemically by in vitro GST pull down assay, eliminating that binding differences to caspase 1 are responsible for this result (Figure 7). Ectopic expression of ASC resulted in the formation of perinuclear aggregates, while deletion of the linker prevented these aggregates and forced ASC-b to the cytosol. However, co-expression with constitutively active NLRP3^R260W ^or full-length ASC, but not caspase 1 was able to restore aggregate formation, suggesting that the linker is essential for self-oligomerization of ASC, but that ASC-b retains the ability to oligomerize with ASC, NLRs and caspase 1 into inflammasomes. There is currently no indication that caspase 1 itself would cause oligomerization of ASC and caspase 1 activation. The proposed mechanism suggests that NTP-mediated NLR oligomerization causes aggregation of ASC and clustering of caspase 1, followed by activation of caspase 1 by induced proximity, and our results suggest that NLRs cause ASC aggregation even in the absence of ASC self-aggregation. We performed this assay in the presence of a constitutive active NLRP3 mutant to trigger assembly of inflammasomes, which allowed us to transfect very low concentrations of ASC to prevent any effects resulting from self-oligomerization of ASC in this assay. In addition, stable expression of ASC or ASC-b in RAW264.7 macrophages resulted in low ASC expression and inflammasomes were activated using LPS/ATP, which has been recently shown by others and us to cause aggregation of ASC, NLRP3 and caspase 1 [37,57]. In this system, the lower activity of ASC-b is consistent with a recent analysis of the structure of intact ASC, which concluded that the linker region, which is absent in ASC-b, is required for providing the necessary degree of flexibility for ASC to facilitate the PYD and CARD interactions essential for inflammasome formation [58]. Thus, lack of this flexible linker would significantly impair the capability of ASC to simultaneously interact with NLRs and caspase 1, resulting in reduced maturation of IL-1β, which is consistent with our observation. Nevertheless, ASC-caspase 1 oligomers in the absence of NLRs have been implicated in pyroptosis, suggesting that ASC-b might likely prevent pyroptosis as well, while still being able to promote IL-1β and IL-18 maturation [59]. Thus, recruitment of ASC-b to pyroptosomes might be another mechanism aside from quickly releasing caspase 1 into the culture medium to prevent inflammation-induced cell death. In addition, since ASC-b is cytosolic, either the linker is directly responsible for nuclear import of ASC or some degree of flexibility provided by the linker is required for nuclear localization of ASC.

Ectopic expression of ASC-c resulted in the formation of long cytosolic filamentous structures, as shown for other proteins consisting of only a death domain fold, including ASC-CARD [48,60]. Thus, formation of these structures was consistent with the domain structure of ASC-c, which possessed a severely truncated PYD connected by the linker to the CARD. As predicted, ASC-c retained its ability to co-localize with caspase 1, which was also recruited into the filaments, while it was not capable of co-localizing with active NLRP3^R260W^, which would require an intact PYD. ASC-c was also able to co-localize with full-length ASC, likely, because ASC can dimerize via CARD [48]. ASC-c is essentially comprised of a CARD and might represent another member of the natural caspase 1 inhibitory CARD-only protein (COP) family [36]. Consistently, recruitment of ASC-c significantly impaired inflammasome assembly and prevented maturation of IL-1β, due to lack of an intact PYD, which is required to bridge NLRs with caspase- 1 in inflammasome reconstitution assays as well as following stable expression in LPS-activated human monocytic THP-1 cells and mouse J774A1 macrophages, indicating that in contrast to ASC and ASC-b, ASC-c appears to function as an inhibitory protein. ASC-c is low abundantly expressed in THP-1 cells, but is highly expressed in mouse J774A1 cells, where it potentially might offset the lack of CARD-only proteins in mice [36]. We did not detect ASC-c under the tested conditions in PBM, but primary human macrophages express significant lower ASC levels compared to THP-1 cells, and thus ASC-c might have been below our detection limit.

The lack of any conserved domain in ASC-d suggests that it is not functional as inflammasome adaptor. ASC-d did neither co-localize with caspase 1 nor with active NLRP3^R260W^, suggesting that the portion of the PYD expressed was insufficient to mediate a stable PYD-PYD interaction. Current models predict PYD-PYD interactions utilizing surfaces on either α-helices 2 and 3 or 1 and 4, suggesting that the α-helices 1 and 2 present in ASC-d are insufficient to mediate PYD-PYD interactions [61]. The interaction between ASC and NLRP3 likely occurs at a positive electrostatic potential surface (EPSP) patch on the PYD of ASC formed by Lys^21^, Lys^22^, Lys^26 ^and Arg^41^, and a negative EPSP on NLRP3, and ASC-d lacks Arg^41 ^[62]. We also could not detect endogenous ASC-d by immunoblot, although it should be detectable by ASC antibodies recognizing the PYD between amino acids 1 to 37. One explanation might be that ASC-d is expressed at very low levels, is not very stable, as indicated from transient expression experiments, is only temporarily expressed, or is not induced by the tested pro-inflammatory stimuli, but rather by anti-inflammatory stimuli during the resolution of inflammatory responses, as recently shown for Nod2. Nod2 functions as a PRR for MDP, while the dominant negative Nod2-S, which has a premature stop in the second CARD due to lack of exon 3, is induced specifically by the anti-inflammatory cytokine IL-10, but repressed by TNF-α and IFN-γ [63]. Alternatively, splicing of ASC-d could be a mechanism to prevent generation of transcripts encoding activating ASC proteins [64]. A similar scenario has been recently proposed for RIP2-β, an alternative splice form of RIP2 lacking any of the known functions of RIP2 [56]. However, ASC-d could have a yet to be identified function, which is in part supported by the unique localization pattern observed in some cells. Nevertheless, due to the low degree of conservation between ASC and ASC-d, the precise relationship, if any, of ASC-d to ASC is currently elusive and will need further investigation.

To gain more insights into the precise expression pattern and function of ASC isoforms, further expression profiling following different cytokine treatment will be needed. One can also assume that different ASC isoforms might likely impact also other functions of ASC, including apoptosis, anoikis, pyroptosis, NF-κB and MAPK activation, however, this will need further studies. Our study revealed the existence of alternative splice variants of ASC, suggesting that the presence of distinct combinations of ASC splice variants might potentially affect inflammasome activity at different stages of the inflammatory response, and further emphasizes the existence of multiple regulatory mechanisms controlling IL-1β and IL-18 processing and release in macrophages and the significance of alternative splicing in fine-tuning the inflammatory host response.

## Conclusions

Generation of different splice variants of ASC potentially provides a mechanism to regulate assembly and activity of inflammasomes and thereby release of IL-1β and IL-18 during the inflammatory host response. Expression of different ratios of activating and inhibitory isoforms of ASC might promote inflammation at early stages of infections and tissue damage, but potentially also allows to terminate these reactions during the resolution phase.

## Competing interests

The authors declare that they have no competing interests.

## Authors' contributions

NBB, AD, SJK and CY carried out the experiments, NBB and AD also drafted the manuscript, YR was involved in the design and interpretation of results, and CS conceived of the study, participated in its design and coordination, performed experiments and helped to draft the manuscript. All authors read and approved the final manuscript.
